# Nekrotisierende Myositis des Oberschenkels: Ursache oder Folge eines systemischen „Capillary-leak“-Syndroms?

**DOI:** 10.1007/s00113-021-01110-7

**Published:** 2021-11-30

**Authors:** Paul Schmitz, Nadine Hausmann

**Affiliations:** Caritas-Krankenhaus St. Josef, Klinik für Unfallchirurgie, Universitätsklinikum Regensburg, Landshuter Str. 65, 93053 Regensburg, Deutschland

**Keywords:** SCLS, Systemisches Capillary-leak-Syndrom, Infektion, Abszess, Kompartmentresektion, SCLS, Systemic capillary leak syndrome, Infection, Abscess, Compartment resection

## Abstract

Infektionen des muskuloskeletalen Systems sind in der Unfallchirurgie mitunter die Krankheitsbilder, die Patienten am gravierendsten beeinträchtigen, Ärzte fachlich sowie Kliniken und das Gesundheitssystem ökonomisch herausfordern. Das *s*ystemische „*C*apillary-*l*eak“-*S*yndrom (SCLS) ist ein seltenes idiopathisches Syndrom, welches auch bei harmlosen Infektionen fulminante lebensbedrohliche Verläufe provozieren kann. Neben einer übersichtlichen Darstellung des SCLS wird über einen betroffenen Patienten, der infolge einer instabilen Narbe eine nekrotisierende Myositis des Oberschenkels entwickelte, berichtet.

## Abstract (en)

Infectious diseases of the musculoskeletal system are in trauma surgery sometimes the symptoms that most severely impair patients and are a challenge to surgeons, hospitals and the economy of the public healthcare system. The systemic capillary leak syndrome (SCLS) is a rare idiopathic syndrome that can provoke a fulminant life-threatening progress even if the primary infection is trivial. In addition to a clearly arranged description of the SCLS we report on an affected patient who developed a necrotizing myositis of the right thigh due to an unstable scar.

Wir berichten über einen 50-jährigen Patienten mit systemischem „Capillary-leak“-Syndrom (SCLS), der aufgrund einer nekrotisierenden Myositis des rechten Musculus (M.) quadriceps femoris behandelt wurde.

## Anamnese

Der Patient wurde aufgrund lokaler Schmerzen im Bereich des rechten Oberschenkels und systemischer Zeichen einer Infektion (Fieber bis 40 °C, Leukozyten 12.000/ml, CRP 2,9 mg/dl [Referenzbereich < 0,5 mg/dl], Hypotonie) stationär vorstellig. Initial erfolgten eine Abszesspunktion mit Spülung und eine systemische Antibiotikatherapie (Piperacillin + Tazobactam). Bei persistierender Infektion erfolgte eine operative Revision mit Lascheneinlage. Hierbei zeigte sich eine ausgedehnte Myositis, woraufhin der Patient zur adjuvanten hyperbaren Oxygenation (HBO) in unsere Klinik für Unfallchirurgie, Caritas Krankenhaus St. Josef – Regensburg, verlegt wurde. Die Anamnese wies bereits 2 schwere Episoden des SCLS nach Chlamydienpneumonie und Influenza-B-Infektion mit Aspirationspneumonie auf. Im Rahmen der vorangegangenen Episode kam es zu einer Hämokonzentration mit Hämoglobin(Hb)-Werten von 21,6 mg/dl, Hämatokrit (HKT) von 65 %, Lactatanstieg (4,9 mmol/l) sowie zu einer ausgeprägten Hypalbuminämie (1930 mg/dl). Darüber hinaus fielen ein Anstieg des IL‑6 und eine IgG_κ_-Paraproteinämie auf. Aufgrund eines Kreislaufversagens erfolgten eine kurzzeitige Reanimation und eine Vasopressortherapie. In den folgenden 4 Tagen kam es zu einem massiven generalisierten Ödem mit Rhabdomyolyse (CK > 10.000U/l), Crush-Niere (Kreatinin > 4 mg/dl) sowie ulzerierenden Spannungsblasen v. a. an den unteren Extremitäten. Differenzialdiagnostisch wurden ein nephrotisches Syndrom, eine Leberzirrhose sowie ein Gleich-Syndrom [4] ausgeschlossen.

**Figure 1:**
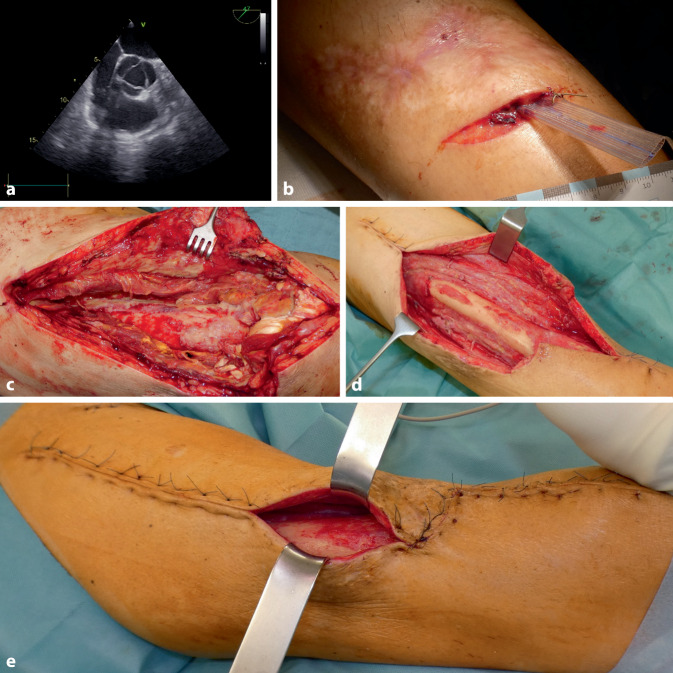

Als Residuen der ersten beiden SCLS-Episoden verblieben eine ausgeprägte „Critical-illness“-Polyneuropathie und Myopathie der Extremitäten sowie Volkmann-Kontrakturen beider Hände. Weiterhin verblieben multiple instabile Narben an Körperstamm und den Extremitäten (Abb. 1b).

## Befund

Bei Aufnahme präsentierte sich der Patient außerhalb des akuten Stadiums einer Sepsis, fieberfrei und hämodynamisch stabil. Der rechte Oberschenkel war stark geschwollen und überwärmt, jedoch nicht gerötet. Zudem zeigte sich eine reizlose Narbe auf Höhe des mittleren Oberschenkels (Abb. 1b). Zwei Querinzisionen ober- und unterhalb der Narbe kommunizierten über eine drainierende Lasche miteinander. Sonographisch zeigte sich ein gekammerter Verhalt im M. quadriceps femoris (Abb. 1a).

Bei persistierenden Entzündungswerten (Leukozyten 10.000/µl, CRP 9,9 mg/dl) und lokalen Infektionszeichen erfolgte die notfallmäßige operative Exploration. Intraoperativ zeigten sich ein ausgedehnter Abszess, eine Infektion der Mm. vastus lateralis et intermedius, durchsetzt mit Nekrosen der Muskulatur und der Faszien (Abb. 1c).

## Diagnose

Nekrotisierende Myositis des rechten M. quadriceps femoris bei vorbestehendem SCLS.

## Therapie

Es erfolgte das radikale chirurgische Débridement mit Entfernung allen avitalen Gewebes, Spülung und temporärer Weichteildeckung mittels „Vacuum Assisted Closure“(VAC)-Therapie (kontinuierlicher Sog 125 mm Hg). Die programmierte „Second-look“-Therapie am Folgetag [6] zeigte einen Progress der demarkierenden Haut- und Muskelnekrosen, sodass das komplette ventrale Kompartment reseziert werden musste (Abb. 1d). Adjuvant erfolgte eine HBO-Therapie. Diese wurde am Aufnahmetag begonnen und aufgrund des schnellen Konsolidierungsprozesses lediglich auf 5 Sitzungen begrenzt.

## Verlauf

Im Rahmen der Kompartmentresektion wurde der Patient kreislaufinstabil (RR = 75/40 mm Hg) und katecholaminpflichtig. Er wurde für 2 Tage intensivmedizinisch behandelt. Es erfolgte die breite antibiotische Abdeckung mit (Piperacillin + Tazobactam, Clindamycin und Penicillin G). Weder ein zu erwartender Albuminabfall noch eine Hämokonzentration wurden in der aktuellen Episode detektiert.

Nach einer weiteren Wundrevision mit lediglich marginalem Débridement der Wundränder konnte der Patient auf die unfallchirurgische Allgemeinstation verlegt werden. Die mikrobiologischen Gewebeuntersuchungen ergaben bei antibiotischer Vorbehandlung keinen Keimnachweis. Histologisch zeigte sich eine nekrotisierende Myositis mit granulozytärem Entzündungsmuster und vereinzelten Nachweisen von grampositiven Haufenkokken. Bei deutlich rückläufigen Entzündungswerten und gutem Lokalbefund wurde die antibiotische Therapie am Tag 10 auf Penicillin deeskaliert. Die vorbestehende Ereignisprophylaxe mit i.v.-Immunglobulinen wurde während des stationären Aufenthalts fortgesetzt.

Nach 2 weiteren VAC-Wechseln erfolgte die Sekundärnaht 18 Tage nach primärem chirurgischen Débridement (Abb. 1e). Nach 26 Tagen stationären Aufenthalts mit u. a. intensiver physiotherapeutischer Behandlung konnte der Patient bei reizlosen Wundverhältnissen, blandem Entzündungslabor und mobil an Unterarmgehhilfen in die Anschlussheilbehandlung entlassen werden.

## Fallanalyse

### Systemisches Capillary-leak-Syndrom

Das SCLS ist ein seltenes idiopathisches Syndrom (weltweit ca. 500 beschriebene Fälle) [1, 3]. Die Patienten entwickeln auch bei harmlosen Infektionen eine systemische Inflammation mit Interleukinausschüttung und gesteigerter Permeabilität der Kapillaren. Klinisch präsentiert sich dies durch ein generalisiertes Ödem aufgrund einer Flüssigkeitsverlagerung in den Extravasalraum mit konsekutiver Hypovolämie. Zudem zeigen sich laborchemisch ein erhöhter HKT und eine Hypalbuminämie [1]. In ca. 95 % der Fälle fällt im Labor eine monoklonale Gammopathie auf [3]. Nichtwenige Patienten werden durch die fulminante Flüssigkeitsverlagerung hämodynamisch instabil. Eine kausale Therapiemöglichkeit besteht nicht, sodass eine symptomatische, intensivmedizinische Therapie erfolgen muss.

Ein spezifisches histopathologisches Bild des SCLS gibt es nicht. Die Schrankenstörung ist nach Abklingen der Akutphase vollständig reversibel [3].

Als Folge der gesteigerten Kapillarpermeabilität kann es an den Extremitäten zu einem Kompartmentsyndrom und einer Rhabdomyolyse kommen [5]. Ein fulminanter Verlauf, eine Fehleinschätzung des Kompartmentstatus oder eine insuffiziente Therapie kann folgenschwere Störungen der Muskelfunktion und der Propriozeption mit hieraus resultierender lebenslanger Beeinträchtigung des Patienten mit sich bringen.

Patienten mit einem SCLS erleiden im Rahmen von erneuten Infekten häufig Rezidive (Median 0,46/Jahr) [5]. Eine Ereignisprophylaxe mit i.v.-Immunoglobulin, Terbutalin oder Aminophyllin verbessert die Prognose bei SCLS deutlich [2]. Dennoch liegt die 5‑Jahres-Überlebensrate nur bei 73 % [5].

Im aktuellen Fall ist die nekrotisierende Myositis am ehestens auf eine chronische subakute Infektion infolge der instabilen Narbe am Oberschenkel zurückzuführen. Aufgrund der ergriffenen chirurgischen, intensivmedizinischen und adjuvant therapeutischen Maßnahmen konnte eine erneute SCLS-Episode verhindert werden. Die kurzzeitige Katecholaminpflichtigkeit ist am ehesten auf die Kompartmentresektion mit dem damit einhergehenden Volumenverlust zurückzuführen. Dennoch ist zu betonen, dass das radikale chirurgische Débridement die Therapie der Wahl zur Eindämmung einer nekrotisierenden Infektion von Muskeln und Faszien ist. Dieses muss zum Schutz des Gesamtorganismus immer bis ins gesunde Gewebe erfolgen, auch wenn dies einen Funktionsverlust bedeutet. Wenn auch ein Abszess durch Inzision und Einlage einer Lasche primär entlastet werden kann, so ist immer eine Exploration der Abszesshöhle notwendig, um nekrotisierende Prozesse zu detektieren und adäquat zu therapieren.

Adjuvant zur chirurgischen Sanierung ist eine HBO-Therapie bei nekrotisierenden Infektionen indiziert. Durch die maximale Anreicherung von physikalisch gelöstem Sauerstoff im Gewebe werden Anaerobier abgetötet. Weiterhin erfolgt eine Stimulation der neutrophilen Leukozyten und der Fibroblasten. Darüber hinaus führt die verbesserte Oxygenierung des kritisch perfundierten Gewebes zu einer schnelleren Regeneration [7].

## Fazit für die Praxis

- Das systemische „Capillary-leak“-Syndrom (SCLS) ist ein seltenes, lebensbedrohliches Syndrom ohne spezifisches histopathologisches Bild, welches auch bei harmlosen Infektionen zu massiven Volumenverschiebungen in den Extravasalraum führen kann.
- Ein fulminanter Verlauf des SCLS, eine Fehleinschätzung des Kompartmentstatus oder eine insuffiziente Therapie kann folgeschwere Störungen der Muskelfunktion und der Propriozeption mit sich bringen.
- Das radikale chirurgische Débridement bis ins gesunde Gewebe ist die Therapie der Wahl bei nekrotisierenden Infektionen.
- Die hyperbare Oxygenation (HBO) hat einen hohen Stellenwert als adjuvante Therapie bei nekrotisierenden Infektionen.
